# Examining the differences between how doctors and artificial intelligence chatbots handle patient symptoms

**DOI:** 10.1097/JS9.0000000000000565

**Published:** 2023-06-22

**Authors:** Ruhul Amin, Ronald Darwin, Biplab Kumar Dey, Kuldeep Dhama, Talha Bin Emran

**Affiliations:** a Faculty of Pharmaceutical Science, Assam down town University, Panikhaiti, Gandhinagar, Guwahati, Assam; b School of Pharmaceutical Sciences, Vels Institute of Science Technology & Advanced Studies, Chennai; c Division of Pathology, ICAR-Indian Veterinary Research Institute, Bareilly, Uttar Pradesh, India; d Department of Pharmacy, BGC Trust University Bangladesh, Chittagong; e Department of Pharmacy, Faculty of Allied Health Sciences, Daffodil International University, Dhaka, Bangladesh

## Introduction

Seeking medical counsel while experiencing worrying symptoms is essential for accurate diagnosis and treatment. Patients may now choose between human doctors and chatbots powered by artificial intelligence (AI), thanks to the proliferation of digital health services. The purpose of this essay is to evaluate how human doctors and AI chatbots handle patient complaints^1^.

## Response from doctors

Because of their training and experience, doctors know how to provide patients the most accurate assessment of their symptoms. When making a diagnosis, they take into consideration the patient’s medical history, physical examination, and test results.

The doctors who responded stressed the need of a tailored strategy. They pay close attention to the patient as they describe their symptoms, follow-up with pertinent inquiries, and consider any other circumstances that may be relevant to the patient’s condition. They use their knowledge and experience to provide recommendations and suggest courses of action that are specifically designed for the individual patient.

Doctors are there for their patients in more ways than one. They have compassion for their patients and are able to reassure them, since they understand the worries that accompany symptom experience. Concerns may be allayed, symptom origins explored, and next steps in diagnosis and treatment outlined.

## AI answers in chats

Algorithms and machine learning fuelling AI chatbots have the potential to give immediate, automated replies to patient symptoms. They examine extensive medical databases to find a probable explanation or advice by matching the symptoms to predetermined patterns or decision trees^2^.

Artificial intelligence chatbots are capable of providing instantaneous replies and fielding many enquiries at once. They provide standardized data on the basis of medical data-derived statistical probability. Many prevalent disorders are linked to specific symptoms, so chatbots may make educated guesses about possible causes and broad advice for managing symptoms^3^.

But there are restrictions on what kinds of questions an AI chatbot can answer. They may not be able to take into consideration the subtleties of specific patients’ conditions or appreciate the complexities of their symptoms. Unfortunately, correct diagnosis typically requires a physical examination or the ordering of diagnostic tests, neither of which these people can do. They may also not have access to the whole medical history of the patient, which might affect the reliability of their assessments^4^.

## Response analysis

Responses to patients’ symptoms must be accurate, reliable, and patient-specific. Physicians are trained to take into account several indicators while making a diagnosis, such as patient history, physical examination findings, and diagnostic test results. They provide recommendations and treatment strategies that are uniquely developed for each patient Table 1.

**Table 1:** Comparison of physician and AI chatbot responses to patient symptoms.

Patient symptoms	Physician response	AI chatbot response
Patient: I have been experiencing persistent headaches.	Persistent headaches can have various causes, such as tension headaches, migraines, sinusitis, or underlying health conditions. It is important to consult with a healthcare professional who can evaluate your symptoms, perform a physical examination, and recommend appropriate diagnostic tests ^3^.	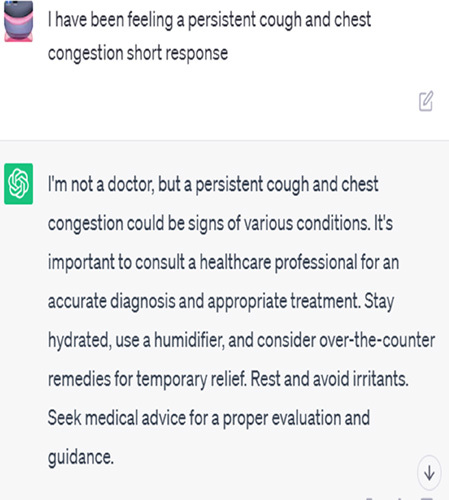
Patient: I have noticed a rash on my skin that is itchy and spreading.	A rash that is itchy and spreading can have several causes, including allergic reactions, skin infections, or dermatological conditions. It is advisable to consult a dermatologist for a proper evaluation, as they can examine the rash and provide an accurate diagnosis and appropriate treatment.	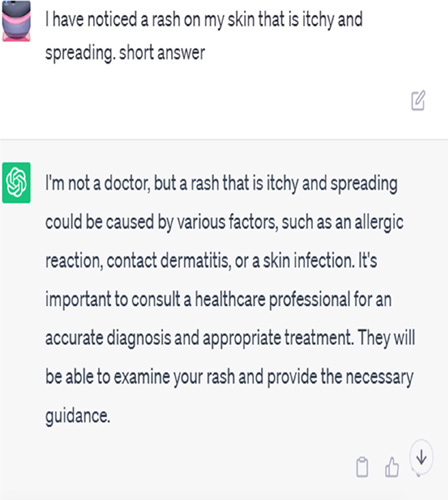
Patient: I have been feeling a persistent cough and chest congestion	A persistent cough and chest congestion can be indicative of various conditions, such as respiratory infections, allergies, or even underlying lung conditions. It is important to consult a pulmonologist who can evaluate your symptoms, listen to your lungs, and recommend further tests or treatments as necessary.	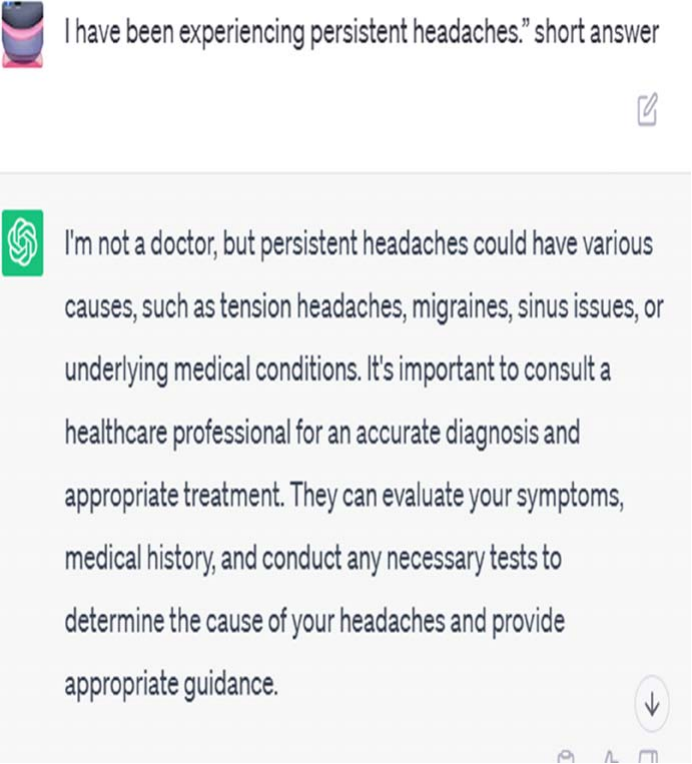

Doctors are also experts at gaining their patients’ trust and emotional support. They may help patients feel heard and understood by addressing their worries, providing answers, and guiding them through the diagnostic procedure. The personal touch and compassion shown by doctors are crucial to the healing process.

Conversely, AI chatbots may be helpful for basic triage and general health information because of their speedy answers. They may utilize statistics to provide insight into the causes of symptoms. Their advice should be taken more as a starting point for further investigation than as a final diagnosis.

## Conclusion

Doctors’ knowledge and attention to detail are irreplaceable when it comes to treating patients’ ailments. AI chatbots cannot replace doctors because they lack the depth of knowledge, clinical expertise, and interpersonal skills that doctors bring to the table. Accurate diagnosis and treatment are guaranteed by their capacity to think about the patient’s medical history, conduct physical examinations, and order diagnostic testing.

AI chatbots may be convenient for their speed and broad insights, but they are no match for a human doctor’s in-depth knowledge and individualized attention. Patient happiness and health depend critically on the human aspect of healthcare, which includes things like empathy, emotional support, and the capacity to adjust to each individual’s circumstances.

Artificially intelligent chatbots may one day play a role in the provision of rudimentary information and direction as technology progresses. Patients must see competent doctors for proper diagnosis, individualized treatment programs, and the kind attention they deserve. The integration of AI technology with medical professionals has the potential to improve patient care by striking a good technological/human balance.

## Ethical approval

Not applicable.

## Source of funding

No funding was received.

## Author contribution

R.A.: conceptualization, data curation, writing—original draft preparation, writing—reviewing and editing, R.D.: data curation, writing—original draft preparation, writing—reviewing and editing. B.K.D.: data curation, writing—original draft preparation, writing—reviewing and editing. K.D.: Writing—reviewing and editing, visualization, supervision. T.B.E.: writing—reviewing and editing, visualization.

## Conflicts of interest disclosure

Authors declare that they have no conflicts of interest.

## Research registration unique identifying number (UIN)

1. Name of the registry: Not applicable.
2. Unique Identifying number or registration ID: Not applicable.
3. Hyperlink to your specific registration (must be publicly accessible and will be checked): Not applicable.

## Guarantor

Talha Bin Emran.

## Data statement

The data in this correspondence article are not sensitive in nature and are accessible in the public domain. The data are therefore available and not of a confidential nature.
